# Efficacy and Safety of Apatinib Combined with Etoposide in Patients with Recurrent Platinum-resistant Epithelial Ovarian Cancer: A Retrospective Study

**DOI:** 10.7150/jca.45547

**Published:** 2020-07-09

**Authors:** Qi Huang, Chaonan Chu, Jie Tang, Zhijie Dai

**Affiliations:** 1 Department of Pharmacy, Hunan Provincial People's Hospital, The First Affiliated Hospital of Hunan Normal University, Changsha, China.; 2 Department of Gynecologic Oncology, Hunan Cancer Hospital, The Affiliated Cancer Hospital of Xiangya School of Medicine, Central South University, Changsha, China.; 3 National Clinical Research Center for Metabolic Diseases, Department of Metabolism and Endocrinology, The Second Xiangya Hospital of Central South University, Changsha, China.

**Keywords:** Apatinib, Etoposide, Epithelial ovarian cancer, Efficacy, Safety

## Abstract

**Purpose:** Advanced epithelial ovarian cancer (EOC) eventually develops into a recurrent platinum-resistant disease. The response to standard treatment and prognosis in patients with EOC is generally unsatisfactory. This study aimed to assess the efficacy and safety of apatinib combined with etoposide in patients with recurrent platinum-resistant EOC.

**Materials and Methods:** This is a single-center, retrospective, observational study. We have reviewed a total of 33 patients with recurrent platinum-resistant EOC from July 2017 to July 2018, who were regularly treated with apatinib and etoposide until disease progression or unacceptable toxic effects occurred.

**Results:** At the date of the review finished, 15 of 33 (45.5%) patients remained on the combined treatment of apatinib and etoposide, while the other 18 (54.5%) had discontinued. Although no complete response (CR) occurred, the overall response rate (ORR) and disease control rate (DCR) were 36.4% and 78.8% respectively. The median progression-free survival (PFS) was 5.6 months (95% CI, 4.1~7.1), and the median overall survival (OS) was 10.3 months (95% CI, 9.4~11.2). The most common adverse event was mucositis oral (60.6%), which caused the treatment discontinued in 4 (12.1%) patients. Other relatively common adverse events were hand-foot syndrome (42.4%), hypertension (39.4%), nausea or vomiting (30.3%), neutropenia (24.2%), fatigue (24.2%) and thrombocytopenia (21.2%). Grade 1 and 2 adverse events accounted for 63.6% (21/33).

**Conclusion:** The efficacy of apatinib combined with etoposide is encouraging in patients with platinum-resistant EOC. Most adverse events of this combined therapy were mild and tolerable. Severe mucositis oral was not rare, which needs more precautions.

## Introduction

Ovarian cancer is the most common cause of gynecological cancer-associated death. Over 295,000 women developed epithelial ovarian cancer (EOC) worldwide in 2018, of whom over 184,000 died 1. Approximately 70% to 80% of patients underwent advanced EOC due to no noticeable and specific symptoms of early ovarian cancer 2. Although surgery and platinum-based cytotoxic chemotherapy can be curative for patients with EOC at an early stage, most women with advanced EOC develop episodes of recurrences with the progressively shorter disease-free interval 3 and platinum resistance 4. Patients with platinum-resistant ovarian cancer showed lower response rates of platinum or non-platinum drugs than platinum-sensitive patients did, whose prognosis was also poorer.

Angiogenesis is a significant factor in the oncogenesis, development, and metastasis of malignant tumors 5. Apatinib, a targeting inhibitor of vascular endothelial growth factor receptor 2 (VEGFR2), is independently researched and developed as an anti-angiogenic drug in mainland China. It was approved in the treatment of advanced gastric cancer and gastroesophageal junction adenocarcinoma by the National Medical Products Administration (NMPA) in China. Apatinib also shows antitumor activities in several other malignant tumors, such as lung cancer, liver cancer, and ovarian cancer 6-9. In a previous multi-center phase II study, 29 patients of platinum-resistant EOC accepted apatinib monotherapy, with the OS of 41.4% (95% CI, 23.3%~59.4%) and the median PFS of 5.1 months (95% CI, 3.8~6.5) 10. No complete response (CR) case of platinum-resistant EOC has been reported yet, and the prognosis is still not up to expectation.

Anti-angiogenesis therapy combined with single-agent chemotherapy has been proved to be associated with a better clinical outcome in patients with ovarian cancer 11. Etoposide is a topoisomerase inhibitor that suppresses the activity of DNA topoisomerase II and forms a stable complex of DNA-TopoII-EP. It executes cytotoxicity by fragmenting DNA double-strand structure and is applicable in the treatment of recurrent ovarian cancer 12. It was reported that the overall response rate and clinical benefit rate of oral etoposide were 19.2% and 40.4% in 52 platinum-resistant EOC patients, respectively 13. However, 13.4% of patients suffered grade 3~4 haematologic or non-haematologic adverse events, indicating slightly high toxicity of etoposide. The combined therapy of apatinib and etoposide was hence investigated in patients with platinum-resistant EOC for higher efficacy and better safety. Furthermore, since both apatinib and etoposide have oral preparations, this combination is an ideal option for patients who prefer home administration rather than hospitalization. In this retrospective study, we reviewed patients who were diagnosed as recurrent platinum-resistant EOC and treated with apatinib combined with etoposide. Based on the observation, we analyzed the efficacy and safety of the combined apatinib and etoposide treatment.

## Methods

### Patients

Patients with recurrent platinum-resistant EOC were reviewed from July 2017 to July 2018 in Hunan Cancer Hospital. Eligibility criteria included age in the range between 18 and 70; diagnosis histologically or cytologically confirmed as EOC; patients developing progressive EOC after cytoreductive surgery and combined chemotherapy; patients resistant to the first-line chemotherapy; at least one measurable solid tumor (> 10 mm) used for efficacy evaluation; Eastern Cooperative Oncology Group (ECOG) performance status 0-2. Exclusion criteria included a diagnosis of other malignancies; previous exposure to the combination of apatinib and etoposide; Karnofsky score below 60; significant abnormality in routine blood test (including neutropenia, thrombocytopenia, and anemia), hepatic and renal function examinations, or electrocardiogram; pregnant and lactating patients. This study has been approved by the Institutional Ethics Committee of Hunan Cancer Hospital and was performed following the Declaration of Helsinki. All patients provided written informed consent before the therapy procedures.

### Treatment

Patients were treated with oral apatinib at an initial dose of 500 mg once daily and oral etoposide at a dose of 50 mg once daily on days 1 to 21 of a 28-day cycle. Dose reductions and interruptions were allowed to handle adverse events. When grade 3 or 4 toxicities occurred, apatinib was reduced to 250 mg once daily. Unless recovery to grade 1 or better non-haematological toxicities and grade 2 or better haematological toxicities, apatinib would not be resumed at its initial dosage. For all grade 3 or 4 toxicities (except those caused by apatinib, such as mucositis oral, hypertension, and hand-foot syndrome), etoposide was withheld until recovery to grade 1 or below non-haematological and grade 2 or below haematological toxicities. Then etoposide was resumed at the initial dose. If toxicities were still intolerable after the dose reduction and interruption or disease progressed, the treatment was terminated.

### Efficacy and safety assessments

The efficacy of the combination therapy was evaluated according to Response Evaluation Criteria in Solid Tumors (RECIST) 1.1, including CR, partial response (PR), stable disease (SD), and progressive disease (PD). Radiologic assessments were conducted by contrast-enhanced CT (CECT) at the baseline and every eight weeks thereafter until progression. Serum CA-125 level, as an adjuvant therapeutic indicator, was detected at the baseline and every four weeks thereafter 14,15. The overall response rate (ORR) was defined as the proportion of patients who achieved CR and PR. Disease control rate (DCR) was defined as the proportion of patients who achieved CR, PR, and SD. Progression-free survival (PFS) was defined as the duration from registration to investigator-assessed disease progression or death, whichever occurred first. Overall survival (OS) was defined as the duration from registration to death. To assess tumor shrinkage, the percentage change in target lesion size was analyzed by investigators using CT scans before and after the treatment. Adverse events were monitored throughout the treatment period and graded following the Common Terminology Criteria for Adverse Events (CTCAE) 4.0.

### Data Analysis

Statistical analyses were performed using Statistical Package for Social Sciences (SPSS) software (Version 17, SPSS, Inc., Chicago, IL, USA). The Kaplan-Meier method was used to create the survival curve.

## Results

### Patient Characteristics

A total of 33 patients with platinum-resistant EOC who were treated with apatinib and etoposide were enrolled in the study. Table 1 shows their clinical characteristics and tumor conditions at the baseline before the administration of apatinib and etoposide. The median age of recurrence was 55 years (range 49~67). The median number of medications in previous chemotherapy was 2 (range 2~5). Most patients (90.9%) had received 2 or 3 medications in previous treatment, and 9.1% of patients had taken more than three medications before apatinib and etoposide were prescribed.

### Efficacy and survival analysis

**Figure 1:**
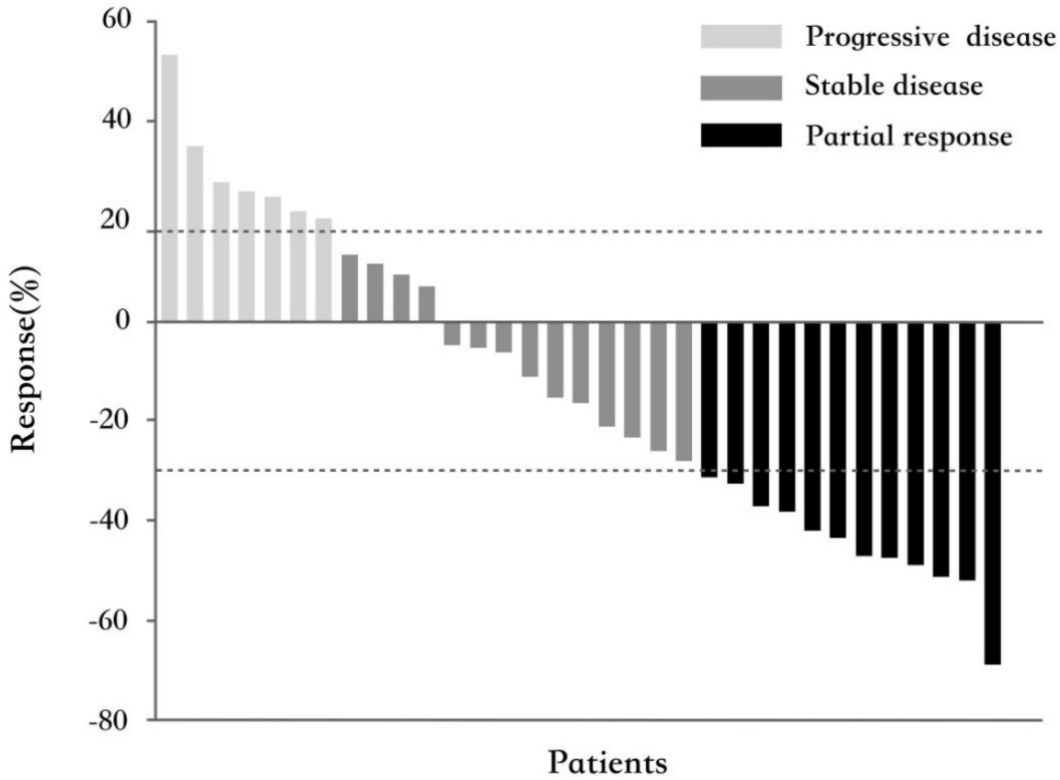
** Waterfall plot for the best percentage change in target lesion size (n=33).** The color indicates the type of response. The dashed lines at 20% and -30% represent the boundary for the determinations of progressive disease and partial response, respectively.

All 33 patients were eligible for response evaluation. Although no CR occurred, PR had been observed in 12 patients (36.4%). The ORR was 36.4%. Their median response duration was 4.9 months (range 2.8~14), and 4 of them (33.3%) had a response duration longer than 6 months. Fourteen patients (42.4%) had experienced SD with the median duration of 4.2 months (range 2.8~12.1), of whom 3 (21.4%) patients had a response duration longer than 6 months. The calculated DCR was 78.8%. Figure 1 shows the best percentage change in tumor size.

**Figure 2:**
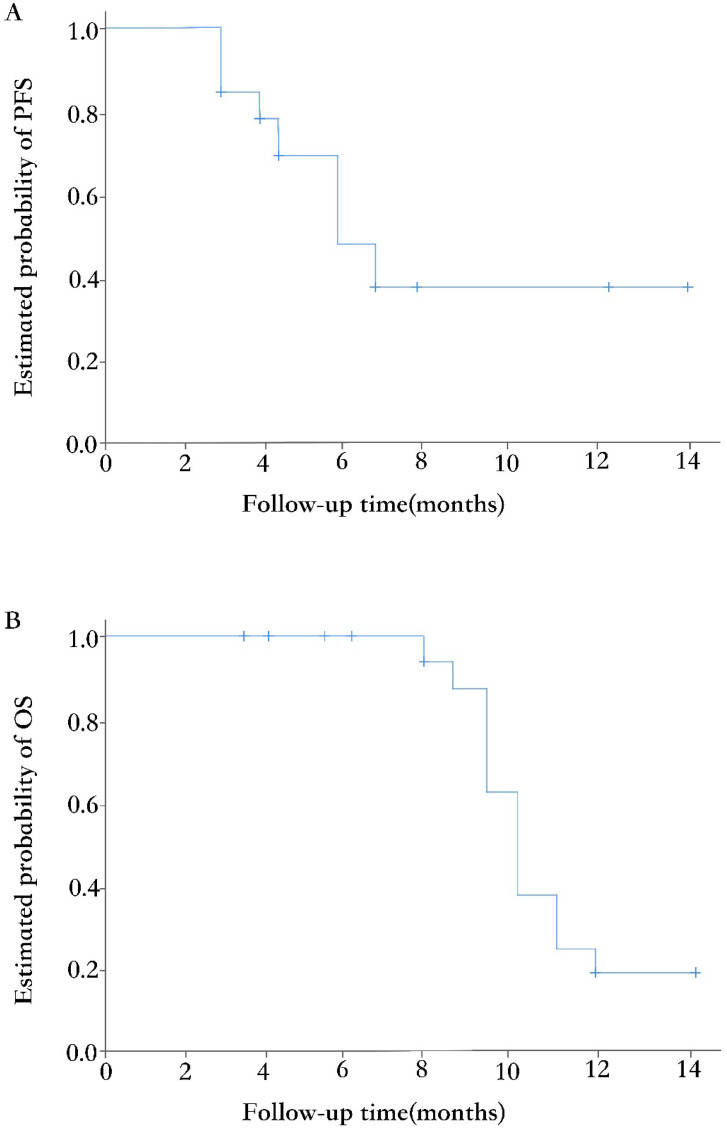
Kaplan-Meier curves of PFS (A) and OS (B).

The median duration of follow-up at the time of data analysis was 7.5 months (range 3.7~14). Eighteen of 33 (54.5%) patients had discontinued the treatment, while the other 15 (45.5%) patients remained on the procedure. The primary reason for treatment discontinuation was disease progression (11 of 18, 61.1%). Other reasons included adverse events (4, 22.2%) and lost to follow-up (3, 16.7%). Fifteen (45.5%) patients had tumors progressed during the treatment combined of apatinib and etoposide, with the median PFS of 5.6 months (95% CI, 4.1~ 7.1; Figure 2A). At the time of analysis, 20 patients were still alive, and the median OS was 10.3 months (95% CI, 9.4~11.2; Figure 2B).

### Safety

All patients experienced one or more treatment-related adverse events (Table 2). Mucositis oral was the most common one, with a high incidence of 60.6%. Four patients were unable to eat normally due to grade 3 or 4 mucositis oral and discontinued the treatment. Other relatively common adverse events included hand-foot syndrome (45.5%), hypertension (39.4%), nausea or vomiting (30.3%), neutropenia (24.2%), fatigue (21.2%) and thrombocytopenia (21.2%). Although 8 patients (24.2%) underwent dose reduction of apatinib because of severe adverse events (grade 3 or 4), the majority of patients (63.6%, 21 of 33) simply experienced mild and controllable adverse events of grade 1 or 2. These adverse events were consistent with those described in the drug specification. No unreported drug adverse reactions have been observed in this study.

## Discussion

Platinum-resistant recurrent ovarian cancer is usually treated with non-platinum or second-line platinum drugs due to its insensitivity to first-line platinum drugs. Angiogenesis is a key mechanism for generation, proliferation, local infiltration, and distant metastasis of tumor cells 16. It is regulated by the interaction between vascular endothelial growth factors (VEGFs) and their receptors (VEGFRs). New drugs or therapeutic strategies targeting neovascularization are of great interest in the management of ovarian cancer. Evidence indicates that blockage of VEGF/ VEGFR pathway by competitively binding to VEGF or interfering with certain domains of VEGFR would be a promising treatment regimen. VEGF-A and VEGFR-2 have been proved to be closely related to the pathological angiogenesis of tumors. Bevacizumab, a recombinant humanized monoclonal IgG1 antibody of VEGF-A, is the first approved anti-angiogenesis drug for ovarian cancer, applicated alone or in combination with cytotoxic chemotherapy. The response rate of bevacizumab in patients with platinum-sensitive or platinum-resistant recurrent ovarian cancer was only acceptable of 20%~30% 4. Application of Bevacizumab also requires frequent hospital visits and long-term intravenous administration, which may decline patients' compliance. Targeting drugs with higher responses and more convenient oral preparation are desired.

Recently, many VEGFR-2 inhibitors have been developed, such as sorafenib, cediranib, and apatinib 17-19. Apatinib is a novel oral angiogenesis inhibitor targeting the intracellular ATP binding site of VEGFR2. It decreases the density of tumor micro-vessels, slows down, and even stops the tumor growth and development by inhibiting VEGFR2 20. Apatinib has been licensed for advanced gastric adenocarcinoma and gastroesophageal junction adenocarcinoma. However, the application of apatinib in ovarian cancer is still in the stage of the clinical study. There are several case reports and clinical studies (n=9~29) focused on apatinib therapy in patients with recurrent platinum-resistant ovarian cancer 8-10,21. As reported in the above literature, the ORR and DCR of apatinib monotherapy could reach 18.2~41.4% and 47.1~81.8% respectively 8-10. The ORR of apatinib monotherapy was higher than that of other single-agent drugs, such as etoposide, vinorelbine, irinotecan, oxaliplatin and gemcitabine, which was only 10~37% 22, 23. However, it is still less than ideal and no CR case has been reported. The reason may be the limited efficacy of apatinib monotherapy. Attempts to combine apatinib with other therapies are performed to achieve higher or even complete responses.

Oral etoposide used to be a classic cost-effective oral medication for patients with recurrent ovarian cancer, with the ORR of 26.8% (CR of 7.3% and PR of 19.5% respectively) shown by a phase II trial (n=41) 24. However, recently its ORR and clinical benefit rate in recurrent ovarian cancer decreased to 19.2% and 40.4% respectively, and no CR case appeared anymore 13. It may be the result of gradually developed resistance of ovarian cancer to etoposide. The addition of anti-angiogenic therapy to chemotherapy has been shown to improve the outcome of patients with platinum-resistant ovarian cancer. Our study demonstrated a promising efficacy of apatinib combined with etoposide in patients with recurrent platinum-resistant ovarian cancer. The ORR and DCR were 36.4% and 78.8% respectively, which were generally higher than those of apatinib monotherapy 9,10. The median PFS was 6 months and the longest PFS surprisingly lasted for 20 months, which were markedly longer than those of apatinib monotherapy (2.2~5.1 months) 8-10. A phase II trial by Lan et al. also reported the combination therapy of apatinib and oral etoposide in patients with platinum-resistant or platinum-refractory ovarian cancer (n=35), with the ORR and DCR as high as 54% and 86% respectively 25. The reason that our study showed similar but less significant efficacy of apatinib combined with etoposide may be the discrepancy in the basic conditions of patients. In our study, 87.8% of patients took the last chemotherapy of non-platinum-based medication, which was much more than those (43%) in Lan's study did. It indicates that our cohort experienced more treatment failures and presented with worse refractory conditions. Our data reconfirmed the ideal therapeutic effect of apatinib combined with oral etoposide for patients with recurrent and refractory platinum-resistant ovarian cancer.

Patients reviewed in this study accepted oral etoposide 50 mg once daily (days 1-21 of a 28-day cycle) as the initial dose, which was a conventional tolerable dosage according to our previous clinical observation. During the whole treatment process, no patient discontinued the treatment because of the adverse events of etoposide. Apatinib is suggested as 500 mg once daily in patients with solid tumors by some clinical trials 26,27. The Chinese clinical consensus of apatinib in the treatment of gastric cancer also recommends apatinib of 500 mg once daily as an initial dose in underweight and feeble female patients, aged patients, and those with a poor hematopoietic capacity of bone marrow. In our study, the initial dose and recommended dose of apatinib were both 500 mg once daily. Most treatment-related adverse events of the combination therapy were mild and could be well managed by suspending the administration, reducing the dose, and treating symptomatically. Eight patients underwent dose modifications because of severe adverse events (grade 3 and 4), and half of them discontinued the treatment due to eating problems caused by severe mucositis oral. Mucositis oral was the most common adverse event with the incidence of 60.6% in our study, while it was less frequent (only 48%) in Lan's study 25. It also seemed more severe in our cohort that three patients underwent severe mucositis oral of grade 4 whereas no patient did in Lan's study 25. Our clinical data show a similarly high incidence of mucositis oral (over 80%) in patients with ovarian cancer after the treatment of apatinib. Even though intensive oral care was given preventively, more than half of patients still developed mucositis oral of grade 2 or higher. More precautions and attention should be taken against severe mucositis oral in patients with the treatment of apatinib.

In conclusion, the combination therapy of apatinib and oral etoposide shows promising efficacy in patients with recurrent platinum-resistant EOC. For most patients, apatinib is a safe oral agent that can be added to current chemotherapies in EOC patients. Only a few patients could not tolerate the toxicity of apatinib, such as severe mucositis oral. Further randomly controlled prospective cohort studies will be performed to provide more evidence for the indication of apatinib in EOC.

## Figures and Tables

**Table 1:** Clinical characteristics of the patients (n=33) at the baseline

Characteristics	n (%) or Median (Range)
Median age, range (years)	55 (49, 67)
**ECOG status**	
0	9 (27.3)
1	21 (63.6)
2	3 (9.1)
FIGO^*^ stage at initial diagnosis	
IIB	1 (3.0)
IIIB	3 (9.1)
IIIC	24 (72.7)
IVB	5 (15.2)
**Tumor grade**	
Well-differentiated	2 (6.1)
Moderately differentiated	6 (18.2)
Poorly differentiated	25 (75.7)
**Number of medications in previous chemotherapy**	
2	25 (75.7)
3	5 (15.2)
>3	3 (9.1)
**The regimen of last chemotherapy**	
Platinum based	4 (12.1)
Non-platinum based	29 (87.9)
**The interval between last chemotherapy and disease progression**	
< 3 months	23 (69.7)
≥3 months and <6 months	10 (30.3)
≥6 months	3 (9.1)

*FIGO: International Federation of Gynecology and Obstetrics.

**Table 2:** Adverse events according to CTCAE 4.0 [n (%)]

Adverse event	Grade 1	Grade 2	Grade 3	Grade 4	Grade 5	Total
Mucositis oral	4 (12.1)	8 (24.2)	5 (15.2)	3 (9.1)	0	20 (60.6)
Hand-foot syndrome	3 (9.1)	6 (18.2)	6 (18.2)	-	-	15 (45.5)
Hypertension	2 (6.1)	8 (24.2)	3 (9.1)	0	0	13 (39.4)
Nausea or vomiting	3 (9.1)	7 (21.2)	0	0	0	10 (30.3)
Neutropenia	7 (21.2)	1 (3.0)	0	0	0	8 (24.2)
Fatigue	3 (9.1)	4 (12.1)	0	-	-	7 (21.2)
Thrombocytopenia	2 (6.1)	4 (12.1)	1 (3.0)	0	0	7 (21.2)
Transaminase increased	3 (9.1)	3 (9.1)	0	0	-	6 (18.2)
Proteinuria	5 (15.2)	0	0	-	-	5 (15.2)
Pain	2 (6.1)	1 (3.0)	0	-	-	3 (9.1)
Diarrhea	0	2 (6.1)	0	0	0	2 (6.1)
